# Gaussian Process Regression for Data Fulfilling Linear Differential Equations with Localized Sources †

**DOI:** 10.3390/e22020152

**Published:** 2020-01-27

**Authors:** Christopher G. Albert, Katharina Rath

**Affiliations:** 1 Max-Planck-Institut für Plasmaphysik, Boltzmannstr. 2, 85748 Garching, Germany; katharina.rath@ipp.mpg.de; 2 Department of Statistics, Ludwig-Maximilians-Universität München, Ludwigstr. 33, 80539 Munich, Germany

**Keywords:** Gaussian process regression, physics-informed methods, kernel methods, field reconstruction, source localization, partial differential equations, meshless methods

## Abstract

Specialized Gaussian process regression is presented for data that are known to fulfill a given linear differential equation with vanishing or localized sources. The method allows estimation of system parameters as well as strength and location of point sources. It is applicable to a wide range of data from measurement and simulation. The underlying principle is the well-known invariance of the Gaussian probability distribution under linear operators, in particular differentiation. In contrast to approaches with a generic covariance function/kernel, we restrict the Gaussian process to generate only solutions of the homogeneous part of the differential equation. This requires specialized kernels with a direct correspondence of certain kernel hyperparameters to parameters in the underlying equation and leads to more reliable regression results with less training data. Inhomogeneous contributions from linear superposition of point sources are treated via a linear model over fundamental solutions. Maximum likelihood estimates for hyperparameters and source positions are obtained by nonlinear optimization. For differential equations representing laws of physics the present approach generates only physically possible solutions, and estimated hyperparameters represent physical properties. After a general derivation, modeling of source-free data and parameter estimation is demonstrated for Laplace’s equation and the heat/diffusion equation. Finally, the Helmholtz equation with point sources is treated, representing scalar wave data such as acoustic pressure in the frequency domain.

## 1. Introduction

The larger context of the present work is the goal to construct reduced complexity models as emulators or surrogates that retain mathematical and physical properties of the underlying system. In recent terminology, such approaches are examples of “physics informed machine learning”. Similar to usual numerical models, the aim here is to represent infinite systems by exploiting finite information in some optimal sense. In the spirit of structure preserving numerics, one tries to move errors to the “right place” to retain laws such as conservation of mass, energy, or momentum. Here, we treat data known to fulfill a given linear differential equation. This article is an extended version of a conference paper [1] presented at the MaxEnt workshop 2019. The revised text adds hyperparameter optimization, results for the heat equation and detailed comparisons to existing methods.

This article deals with Gaussian process (GP) regression on data with additional information known in the form of linear, generally partial differential equations (PDEs). An illustrative example is the reconstruction of an acoustic sound pressure field and source parameters from discrete microphone measurements. GPs, a special class of random fields, are used in a probabilistic rather than a stochastic sense: estimate a fixed but unknown field from possibly noisy local measurements. Uncertainties in this reconstruction are modeled by a normal distribution.

Using GPs to fit data from PDEs has been a topic of research for some time, especially in the field of geostatistics [2]. A general analysis for deterministic source densities including a number of important properties is given by [3]. In these earlier works GPs are usually referred to as “Kriging” and covariance functions/kernels as “covariograms”. A number of more recent works from various fields [4,5,6,7,8] use the linear operator of a PDE to relate the kernels of source and response field. One of the two is usually modeled by a generic squared exponential kernel. Although the authors of [4,6,7] use such a kernel for the response field and a kernel modified by a differential operator for the source field, [5] models the source field by a generic kernel and applies the inverse (integral) operator to obtain a kernel for the measured response. In contrast to the present approach such methods are suited best for source fields that are non-vanishing across the whole domain. In terms of deterministic numerical methods, one could say that these approaches with volumetric charge densities correspond to meshless variants of the finite element method (FEM).

The approach in the present work instead relies on Gaussian processes that generate *exact* solutions of the homogeneous part of the differential equation [9,10,11]. This is efficient for problems with mostly source-free domains and requires specialized kernels where possible singularities (virtual sources) are moved outside the domain of interest. In particular, boundary conditions on a finite domain can be either supplied or reconstructed in this fashion. Localized internal point sources are then superimposed as a linear model, using again fundamental solutions in the free field. One can thus interpret this approach as a probabilistic variant of a procedure related to the boundary element method (BEM), known as the *method of fundamental solutions (MFS*) or regularized BEM [12,13,14]. As in the BEM, the MFS also builds on fundamental solutions, but allows to place sources outside the boundary rather than localizing them on a layer. Thus, the MFS avoids singularities in boundary integrals of the BEM, while retaining a similar ratio of numerical effort and accuracy for smooth solutions. To the best of the author’s knowledge, the probabilistic variant of the MFS via GPs has first been introduced by [9] to solve the boundary value problem of the Laplace equation and dubbed *Bayesian boundary elements estimation method ((BE)*^2^*M)*. This work also provides a detailed treatment of kernels for the 2D Laplace equation. A more extensive and general treatment of the Bayesian context as well as kernels and their connection to fundamental solutions is available in [10] under the term *probabilistic meshless methods (PMM)*.

Although the authors of [9] treat boundary data of a the homogeneous Laplace equation and the authors of [10] provides a detailed mathematical foundation, the present work aims to extend the recent work on added point sources in [11], unify the derivation of specialized kernels, and demonstrate usefulness in applications. First, a general derivation is given on how to model PDE data by superposing a GP and a linear model for localized sources. Then, the construction of kernels for the homogeneous part of partial differential equations via according fundamental solutions is described in general. Finally, concrete application examples are given for Laplace/Poisson, heat/diffusion and Helmholtz equation for which the derivation of several kernels is presented. Performance is compared to regression with a generic squared exponential kernel, including hyperparameter optimization in all cases. For the Helmholtz equation we estimate strength and positions of sources by nonlinear optimization.

## 2. GP Regression for Data from Linear PDEs

Gaussian process (GP) regression [15] is a tool to represent and update incomplete information on scalar fields u(x), i.e., a real number *u* depending on a (multidimensional) independent variable x (the more general case of complex valued fields and vector fields is left open for future investigations in this context). A GP with mean m(x) and covariance function or kernel $k(x,x^{\prime })$ is denoted as

(1) $$u\left(x\right)∼G(m\left(x\right),k\left(x,x^{\prime }\right)).$$

The choice of an appropriate kernel $k(x,x^{\prime })$ restricts realizations of (1) to respect regularity properties of u(x) such as continuity or characteristic length scales. Often regularity of *u* does not appear by chance, but rather reflects an underlying law. We will exploit such laws in the construction and application of GPs describing *u* for the case described by linear (partial) differential equations:

(2) $$\hat{L}u\left(x\right)=q\left(x\right).$$

where $\hat{L}$ is a linear differential operator and q(x) is a source term. In the laws of physics, dimensions of x usually consist of space and/or time. Physical scalar fields *u* include, e.g., electrostatic potential Φ, temperature *T*, or pressure *p*. Corresponding laws include Gauss’ law of electrostatics for Φ with weighted Laplacian $\hat{L}=\varepsilon \Delta$, thermodynamics for *T* with heat/diffusion operator $\hat{L}=\frac{\partial }{\partial t}-D\Delta$ and frequency-domain acoustics for *p* with Helmholtz operator $\hat{L}=\Delta +k_{0}^{\;2}$. These operators contain free parameters, namely, permeability ε, wavenumber $k_{0}$, and diffusivity *D*, respectively. While ε may be absorbed inside *q* in a uniform material model of electrostatics, estimation of parameters such as *D* or $k_{0}$ is useful for material characterization.

Consider first the source-free (homogeneous) case

(3) $$\hat{L}u_{h}\left(x\right)=0.$$

An unknown field $u_{h}\left(x\right)$ that fulfills (3) shall be modeled by the Gaussian process

(4) $$u_{h}\left(x\right)∼G\left(0,k\left(x,x^{\prime }\right)\right).$$

Application of a linear operator $\hat{L}$ yields a modified Gaussian process

(5) $$\hat{L}u_{h}\left(x\right)∼G\left(0,\hat{L}k\left(x,x^{\prime }\right){\hat{L}}^{\prime }\right),$$

where ${\hat{L}}^{\prime }$ acts from the right side with respect to $x^{\prime }$. In order to fulfill (3) we require (5) to vanish identically, i.e., yield a deterministic zero. Consequently, the kernel $k(x,x^{\prime })$ needs to satisfy

(6) $$\hat{L}k\left(x,x^{\prime }\right){\hat{L}}^{\prime }=0.$$

A discussion on derivation of such kernels is found in Section 2.

For the general case (2), with unknown source density q(x), we introduce a linear model

(7) $$q\left(x\right)=\sum_{i}\varphi_{i}\left(x\right)q_{i}=\varphi^{T}\left(x\right)q,$$

with basis functions $\varphi_{i}\left(x\right)$ and a normally distributed prior

(8) $$\begin{array}{c} q∼N(q_{0},\Sigma_{q}), \end{array}$$

with mean $q_{0}$ and prior covariance $\Sigma_{q}$ for coefficients $q_{i}$ representing source strengths.

For a particulary solution $u_{p}\left(x\right)$ fulfilling the inhomogeneous Equation (2) with source model (8), a linear model induced by the operator $\hat{L}$ follows as

(9) $$u_{p}\left(x\right)=h{\left(x\right)}^{T}q,\;\mathrm{with}\;\hat{L}h_{i}\left(x\right)=\varphi_{i}\left(x\right).$$

Here, coefficients $q_{i}$ remain the same as in (8) and new basis functions $h_{i}\left(x\right)$ fulfil the differential equation with source density $\varphi_{i}\left(x\right)$. In case of point monopole sources $\varphi_{i}\left(x\right)=\delta \left(x-x_{\;i}^{q}\right)$ placed at positions $x_{\;i}^{q}$, each $h_{i}\left(x\right)$ represents a fundamental solution evaluated for the respective source, so

(10) $$h_{i}\left(x\right)=G\left(x,x_{\;i}^{q}\right),$$

where $G(x,x_{\;i}^{q})$ is a Green’s function for operator $\hat{L}$. In the remaining work with localized sources we take this approach. As $G(x,x_{\;i}^{q})$ is usually only available for simple geometries and boundary conditions the discussed linear model alone is limited in its application. We can however represent much more general fields by a superposition of a locally source-free background $u_{h}\left(x\right)$ and point source contributions $u_{p}\left(x\right)$. Boundary conditions induced by external sources are then covered by $u_{h}\left(x\right)$, and internal sources entering $u_{p}\left(x\right)$ are treated via simple free-field Green’s functions. Following the technique of [16] discussed in [15] (Chapter 2.7), the superposition $u\left(x\right)=u_{h}\left(x\right)+u_{p}\left(x\right)$ of the GP $u_{h}\left(x\right)$ and the linear model $u_{p}\left(x\right)$ is distributed according to the Gaussian process

(11) $$u\left(x\right)∼G(h{\left(x\right)}^{T}q_{0},k\left(x,x^{\prime }\right)+h{\left(x\right)}^{T}\Sigma_{q}h\left(x^{\prime }\right)).$$

We will now verify that (11) indeed models the original differential Equation (2) correctly, thereby generalizing the analysis for a deterministic source density in [3]. With $\hat{L}k\left(x,x^{\prime }\right){\hat{L}}^{\prime }=0$, we obtain

(12) $$\hat{L}u\left(x\right)∼G\left(\hat{L}h{\left(x\right)}^{T}q_{0},\hat{L}h{\left(x\right)}^{T}\Sigma_{q}h\left(x^{\prime }\right){\hat{L}}^{\prime }\right)=G\left(\varphi {\left(x\right)}^{T}q_{0},\varphi {\left(x\right)}^{T}\Sigma_{q}\varphi \left(x\right)\right).$$

This is indeed the GP representing the linear source model (8) that we assumed and yields a consistent representation of u(x) and q(x) inside (2).

Using the limit of a vague prior with $q_{0}=0$ and $|\Sigma_{q}^{-1}|\to 0$, i.e., minimum information / infinite prior covariance [15,16], posteriors for mean $\bar{u}$ and covariance matrix cov(u,u) based on given training data $y=u\left(X\right)+\sigma_{n}$ with measurement noise variance $\sigma_{n}^{2}$ are

(13) $$\begin{array}{cc} \bar{u}\left(X_{⋆}\right) & =K_{⋆}^{T}\;K_{y}^{-1}\left(y-H^{T}\bar{q}\right)+H_{⋆}^{T}\bar{q}=K_{⋆}^{T}\;K_{y}^{-1}y+R^{T}\bar{q}, \end{array}$$

(14) $$\begin{array}{cc} \mathrm{cov}(u\left(X_{⋆}\right),u\left(X_{⋆}\right)) & =K_{⋆⋆}-K_{⋆}^{T}\;K_{y}^{-1}K_{⋆}+R^{T}{\left(HK_{y}^{-1}H^{T}\right)}^{-1}R. \end{array}$$

where $X=(x_{1},x_{2},\cdots x_{N})$ contains the training points and $X_{⋆}=\left(x_{⋆1},x_{⋆2},\cdots ,x_{⋆N_{⋆}}\right)$ the evaluation or test points. Functions of *X* and $X^{⋆}$ are to be understood as vectors or matrices resulting from evaluation at different positions, i.e., $\bar{u}\left(X_{⋆}\right)\equiv \left(\bar{u}\left(x_{⋆1}\right),\bar{u}\left(x_{⋆2}\right),\cdots ,\bar{u}\left(x_{⋆N_{⋆}}\right)\right)$ is a tuple of predicted expectation values. The matrix K≡k(X,X) is the covariance of the training data with entries $K_{ij}\equiv k\left(x_{i},x_{j}\right)$. Entries of the predicted covariance matrix for *u* evaluated at the test points $x_{⋆i}$ are $\mathrm{cov}{\left(u\left(X_{⋆}\right),u\left(X_{⋆}\right)\right)}_{ij}\equiv \mathrm{cov}\left(u\left(x_{⋆i}\right),u\left(x_{⋆j}\right)\right)$. Furthermore, $K_{y}\equiv K+\sigma_{n}^{2}I$, $K_{⋆}\equiv k\left(X,X_{⋆}\right)$, $K_{⋆⋆}\equiv k\left(X_{⋆},X_{⋆}\right)$, $R\equiv H_{⋆}-HK_{y}^{-1}K_{⋆}$, and entries of *H* are $H_{ij}\equiv h_{i}\left(x_{j}\right)$, $H_{⋆ij}\equiv h_{i}\left(x_{⋆j}\right)$. Posterior mean and covariance of source strengths are given from the linear model [16] in the limit of a vague prior,

(15) $$\begin{array}{cc} \bar{q} & ={\left(HK_{y}^{-1}H^{T}\right)}^{-1}HK_{y}^{-1}y, \end{array}$$

(16) $$\begin{array}{cc} \mathrm{cov}(q,q) & ={\left(HK_{y}^{-1}H^{T}\right)}^{-1}. \end{array}$$

In the absence of sources, the matrix *R* vanishes, and (13) and (14) reduce to posteriors of a GP with zero prior mean and are directly used to model homogeneous solutions $u_{h}\left(x\right)$ of (3).

### Construction of Kernels for Homogeneous PDEs

For the representation of solutions $u_{h}\left(x\right)$ of homogeneous differential Equations (3), the weight-space view ([15] Chapter 2.1) of Gaussian process regression is useful. There the kernel *k* is represented via a tuple $\phi \left(x\right)=(\phi_{1}\left(x\right),\phi_{2}\left(x\right),\cdots )$ of basis functions $\phi_{i}\left(x\right)$ that underlie a linear regression model

(17) $$u\left(x\right)=\phi {\left(x\right)}^{T}w=\sum_{i}\phi_{i}\left(x\right)w_{i}.$$

Bayesian inference starting from a Gaussian prior with covariance matrix $\Sigma_{p}$ for weights w yields a Mercer kernel

(18) $$k\left(x,x^{\prime }\right)\equiv \phi^{T}\left(x\right)\Sigma_{p}\phi \left(x^{\prime }\right)=\sum_{i,j}\phi_{i}\left(x\right)\Sigma_{p}^{ij}\phi_{j}\left(x^{\prime }\right).$$

The existence of such a representation is guaranteed by Mercer’s theorem in the context of reproducing kernel Hilbert spaces (RKHS) [14]. More generally one can also define kernels on an uncountably infinite number of basis functions in analogy to (17) via

(19) $$f\left(x\right)=\left(\hat{\phi }w\right)\left(x\right)=\langle \phi (x,\zeta ),w(\zeta )\rangle =\int \phi \left(x,\zeta \right)\;w\left(\zeta \right)\;d\zeta ,$$

where $\hat{\phi }$ is a linear operator acting on elements w(ζ) of an infinite-dimensional weight space parametrized by an auxiliary index variable ζ, that may be multidimensional. We represent $\hat{\phi }$ via an inner product $\langle \phi (x,\zeta ),w(\zeta )\rangle$ in the respective function space given by an integral over ζ. The infinite-dimensional analog to the prior covariance matrix is a prior covariance operator ${\hat{\Sigma }}_{p}$ that defines the kernel as a bilinear form

(20) $$k\left(x,x^{\prime }\right)\equiv \langle \phi \left(x,\zeta \right),{\hat{\Sigma }}_{p}\phi \left(x^{\prime },\zeta^{\prime }\right)\rangle \equiv \int \phi \left(x,\zeta \right)\Sigma_{p}\left(\zeta ,\zeta^{\prime }\right)\phi \left(x^{\prime },\zeta^{\prime }\right)\;d\zeta \;d\zeta^{\prime }.$$

Kernels of the form (20) are known as convolution kernels. Such a kernel is at least positive semidefinite, and positive definiteness follows in the case of linearly independent basis functions ϕ(x,ζ) [14].

For treatment of PDEs, the possible choices of index variables in Equation (18) or Equation (20) include separation constants of analytical solutions, or the frequency variable of an integral transform. In accordance with [10], using basis functions that satisfy the underlying PDE, a probabilistic meshless method (PMM) is constructed. In particular, if ζ parameterizes positions of sources, and ϕ(x,ζ)=G(x,ζ) in (20) is chosen to be a fundamental solution/Green’s function G(x,ζ) of the PDE, one may call the resulting scheme a *probabilistic method of fundamental solutions (pMFS)*. In [10], sources are placed across the whole computational domain, and the resulting kernel is called *natural*. Here, we will instead place sources in the exterior to fulfill the homogeneous interior problem, as in the classical MFS [12,13,14]. Technically, this is also achieved by setting $\Sigma_{p}\left(\zeta ,\zeta^{\prime }\right)=0$ for either ζ or $\zeta^{\prime }$ lies in the interior. For discrete sources localized at $\zeta =\zeta_{i}$ one obtains again discrete basis functions $\phi_{i}\left(x\right)=G\left(x,\zeta_{i}\right)$ for (18).

## 3. Application Cases

Here, the general results described in the previous sections are applied to specific equations. First, a specialized kernel fulfilling the given linear differential equation is constructed according to (18), and second, numerical experiments on physical examples are performed comparing the specialized kernel to a squared exponential kernel. Regression is performed based on values measured at a set of sampling points $x_{i}$ and may also include optimization of hyperparameters θ appearing as auxiliary variables inside the kernel $k(x,x^{\prime };\theta )$. The optimization step is, as usually, performed such that the marginal likelihood of the GP is maximized (maximum likelihood or ML values). In the Bayesian sense, this corresponds to a maximum a-posteriori (MAP) estimate for a flat prior. Accordingly, $\theta_{\mathrm{ML}}$ is fixed rather than providing a joint probability distribution function including θ as random variables. We note that depending on the setting this choice may lead to underestimation of uncertainties in the reconstruction of u(x), in particular for sparse, low-quality measurements.

### 3.1. Laplace’s Equation in Two Dimensions

First, we explore construction of kernels fulfilling (5) for a homogeneous problem in a finite and infinite dimensional index space, depending on the mode of separation. Consider Laplace’s equation:

(21) Δu(x)=0.

In contrast to the Helmholtz equation, Laplace’s equation has no scale, i.e., permits all length scales in the solution. In the 2D case using polar coordinates the Laplacian becomes

(22) $$\frac{1}{r}\frac{\partial }{\partial r}\left(r\frac{\partial u(r,\theta )}{\partial r}\right)+\frac{1}{r^{2}}\frac{\partial^{2}u\left(r,\theta \right)}{\partial \theta^{2}}=0.$$

A well-known family of solutions for this problem based on the separation of variables is

(23) $$u\left(r,\theta \right)=r^{\pm m}e^{\pm im\theta },$$

with separation constant *m*, leading to real-valued combinations

(24) $$r^{m}\cos\left(m\theta \right),\;r^{m}\sin\left(m\theta \right),\;r^{-m}\cos\left(m\theta \right),\;r^{-m}\sin\left(m\theta \right).$$

As our aim is to work in bounded regions, we discard the solutions with negative exponent that diverge at r=0. Choosing a diagonal prior that weights sine and cosine terms equivalently [9] and introducing a length scale *ℓ* as a free parameter we obtain a kernel according to (18) with

(25) $$\begin{array}{cc} k(x,x^{\prime };\ell ,\sigma_{m})\; & =\sum_{m=0}^{\infty }{\left(\frac{rr^{\prime }}{\ell^{2}}\right)}^{m}\sigma_{m}^{\;2}\;\left(\cos\left(m\theta \right)\;\cos\left(m\theta^{\prime }\right)+\sin\left(m\theta \right)\;\sin\left(m\theta^{\prime }\right)\right)\\ \; & =\sum_{m=0}^{\infty }{\left(\frac{rr^{\prime }}{\ell^{2}}\right)}^{m}\sigma_{m}^{\;2}\;\cos\left(m(\theta -\theta^{\prime })\right). \end{array}$$

A flat prior $\sigma_{m}^{\;2}=\sigma_{u}^{\;2}$ for all polar harmonics and a characteristic length scale *ℓ* as another hyperparameter yields

(26) $$\begin{array}{cc} k(x,x^{\prime };\ell ,\sigma_{u}) & =\sigma_{u}^{\;2}\frac{1-\frac{rr^{\prime }}{\ell^{2}}\cos\left(\theta -\theta^{\prime }\right)}{1-2\frac{rr^{\prime }}{\ell^{2}}\cos\left(\theta -\theta^{\prime }\right)+\frac{{\left(rr^{\prime }\right)}^{2}}{\ell^{4}}}=\sigma_{u}^{\;2}\frac{1-\frac{x\cdot x^{\prime }}{\ell^{2}}}{1-2\frac{x\cdot x^{\prime }}{\ell^{2}}+\frac{{\left|x\right|}^{2}{\left|x^{\prime }\right|}^{2}}{\ell^{4}}}. \end{array}$$

**Figure 1:**
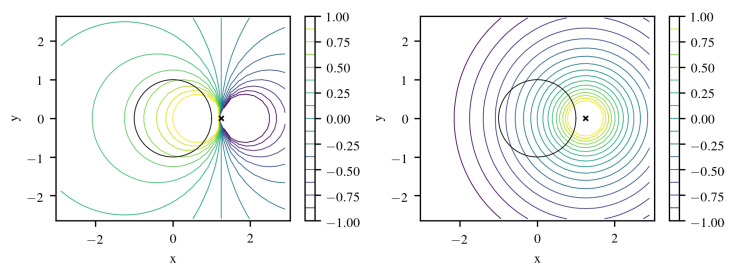
Kernels $k(x,x^{\prime })$ evaluated at x=(x,y) and $x^{\prime }=\left(0.8,0\right)$. **Left**: dipole response of (27), **right**: monopole response of (28). Singularities are moved outside the domain of interest.

This kernel is not stationary, but isotropic around a fixed coordinate origin. Introducing a mirror point ${\bar{x}}^{\prime }$ with polar angle ${\bar{\theta }}^{\prime }=\theta^{\prime }$ and radius ${\bar{r}}^{\prime }=\ell^{2}/r^{\prime }$ we notice that (26) can be written as

(27) $$k\left(x,x^{\prime };\ell ,\sigma_{u}\right)=\sigma_{u}^{\;2}\frac{{\left|{\bar{x}}^{\prime }\right|}^{2}-x\cdot {\bar{x}}^{\prime }}{{\left(x-{\bar{x}}^{\prime }\right)}^{2}},$$

making a dipole singularity apparent at $x={\bar{x}}^{\prime }$. In addition, *k* is normalized to 1 at x=0. Choosing $\ell >R_{0}$ larger than the radius $R_{0}$ of a circle centered in the origin and enclosing the computational domain, we have ${\bar{r}}^{\prime }>\ell^{2}/\ell =\ell >R_{0}$. Thus, all mirror points and the according singularities are moved outside the domain. This behavior is illustrated in Figure 1 where computing the covariance kernel with respect to point $x^{\prime }=\left(0.8,0\right)$ leads to distances >1 everywhere inside the unit circle.

Choosing a slowly decaying $\sigma_{m}^{\;2}=\sigma_{u}^{\;2}/m$, excluding m=1 and adding a constant term yields a logarithmic kernel instead [9] with

(28) $$\begin{array}{cc} k(x,x^{\prime };\ell ,\sigma_{u}) & =\sigma_{u}^{\;2}\left(1-\frac{1}{2}\ln\left(1-2\frac{x\cdot x^{\prime }}{\ell^{2}}+\frac{{\left|x\right|}^{2}{\left|x^{\prime }\right|}^{2}}{\ell^{4}}\right)\right)\\ \; & =\sigma_{u}^{\;2}\left(1-\ln\left(\frac{|x-{\bar{x}}^{\prime }|}{\left|{\bar{x}}^{\prime }\right|}\right)\right). \end{array}$$

Instead of a dipole singularity that expression features a monopole singularity at $x-{\bar{x}}^{\prime }$ that is again avoided by placing it outside the domain for any pair of x and $x^{\prime }$ (Figure 1).

Using instead Cartesian coordinates x,y to separate the Laplacian provides harmonic functions like

(29) $$u\left(x,y\right)=e^{\pm \kappa x}e^{\pm i\kappa y}.$$

Here, all solutions yield finite values at x=0, so we do not have to exclude any of them a priori. Introducing, again, a diagonal covariance operator in (20) and taking the real part yields

(30) k(x,x′;σ2(κ))=∫φ(x,κ)σ2(κ)φ(x′,κ)dκ=Re∫−∞∞σ2(κ)eκ(x±x′)eiκ(y±y′)dκ.

Setting $\sigma^{\;2}\left(\kappa \right)\equiv e^{-2\kappa^{2}}$ and choosing a characteristic length scale *ℓ* together with a possible rotation angle $\theta_{0}$ of the coordinate frame yields the kernel

(31) k(x,x′;ℓ,θ0,σu)=σu22Reexp(x+x′)±i(y−y′)2ei2θ0)ℓ2.

Other sign combinations do not yield a positive definite kernel – similar to the polar kernel (27) before we couldn’t obtain an fully stationary expression that depends only on differences between coordinates of x and $x^{\prime }$.

**Figure 2:**
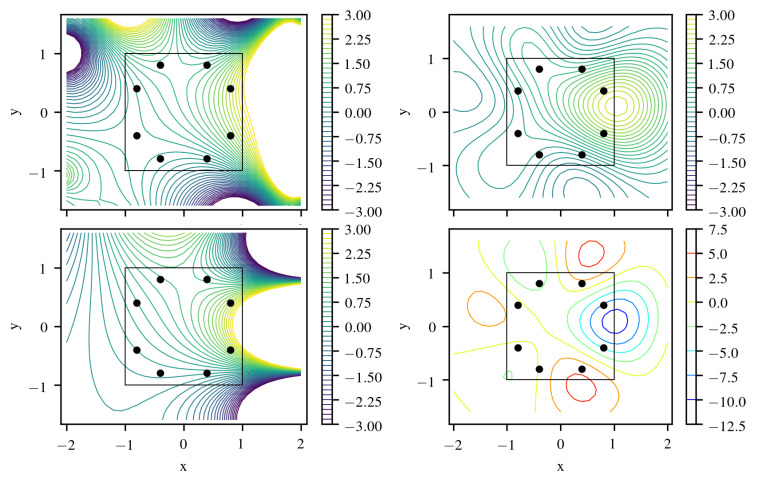
GP reconstruction of Laplace’s equation with specialized locally source-free Mercer kernel (27) (**top left**) and generic squared exponential kernel (**top right**). Sources lie outside the black square region and 8 measurement positions are marked by black dots. Reference analytical solution (**bottom left**). Source density $\bar{q}=\Delta \bar{u}$ of prediction via a generic squared exponential kernel (**bottom right**).

For demonstration purposes we consider an analytical solution to a boundary value problem of Laplace’s equation on a square domain Ω with corners at (x,y)=(±1,±1). The reference solution is

(32) $$u_{\mathrm{ref}}\left(x,y\right)=\frac{1}{2}\left(e^{y}\cos\left(x\right)+e^{2x}\cos\left(2y\right)\right)$$

and depicted in the upper left of Figure 2 together with the extension outside the boundaries. This figure also shows results from a GP fitted based on data with artificial noise of $\sigma_{n}=0.1$ measured at 8 points using kernel (27) with optimized maximum-likelihood (ML) values for hyperparameters *ℓ* and $\sigma_{u}$ but fixed $\sigma_{n}$. Inside Ω the solution is represented with errors below 5%. This is also reflected in the error predicted by the posterior variance of the GP that remains small in the region enclosed by measurement points. The analogy in classical analysis is the theorem that the solution of a homogeneous elliptic equation is fully determined by boundary values.

**Figure 3:**
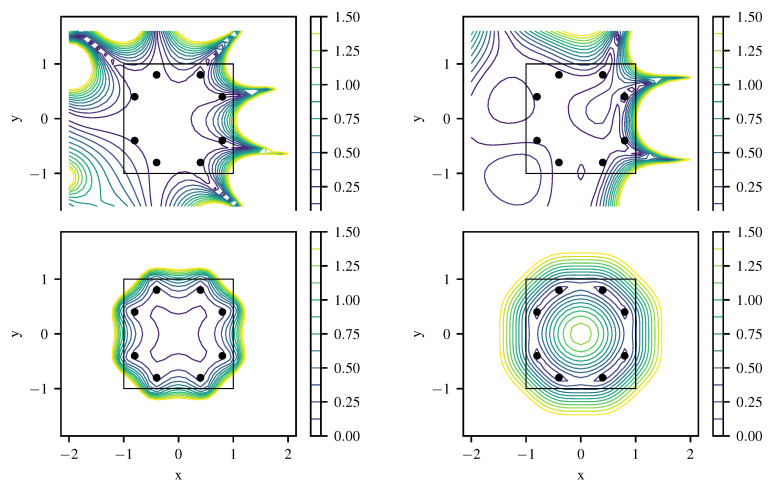
Absolute error (**top left**) and predicted 95% confidence interval (**bottom left**) with specialized locally source-free Mercer kernel (27) in comparison to absolute error (**top right**) and predicted 95% confidence interval (**bottom right**) with generic squared exponential kernel for 8 training points.

**Figure 4:**
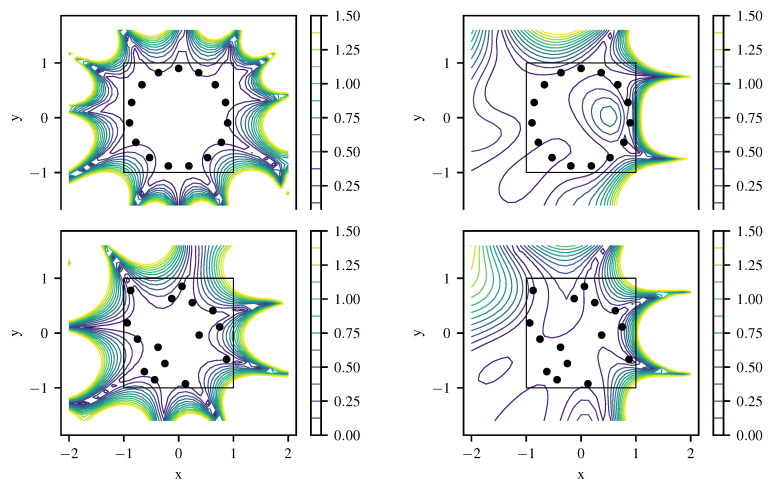
Absolute error as in Figure 3 for 15 training points on a circle (**top**) and for quasi-random points (**bottom**). As the generic squared exponential kernel does not fulfill the given differential equation, even for a larger number of training points, the source density doesn’t vanish in the domain.

**Figure 5:**
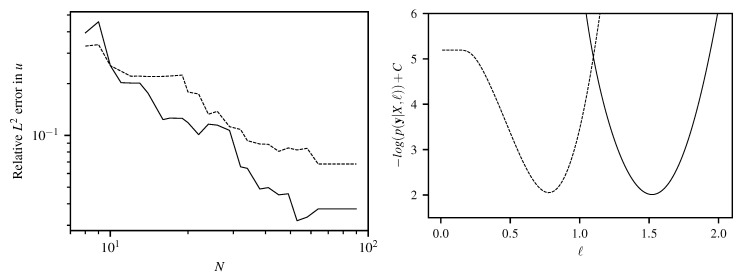
(**Left**) Comparison of relative $L^{2}$ error in *u* for specialized kernel (solid line) and squared exponential kernel (dashed line) for Laplace’s equation for *N* quasi-random training points. (**Right**) Negative log likelihood from 8 training data of Figure 2 with optimum at ℓ=1.52 for specialized kernel (solid line) and at ℓ=0.78 for the squared exponential kernel (dashed line).

In comparison, a reconstruction using a generic squared exponential kernel

(33) $$k\left(x,x^{\prime };\ell ,\sigma_{u}\right)=\sigma_{u}^{2}\exp\left(\frac{-{\left(x-x^{\prime }\right)}^{2}}{2\ell^{2}}\right)$$

yields a much worse approximation quality in Figure 2 and Figure 3. This is in contrast to earlier investigations [1] where a fixed length scale hyperparamter ℓ=2 was used. Although the specialized GP with kernel (27) could identify this length scale during hyperparameter optimization, using a generic kernel (33) leads to an underestimation of *ℓ* and requires twice the number of training points to achieve a similar fit quality and profits from scattered training points, as it has no information about the nature of the boundary value problem (Figure 4 and Figure 5).

In addition, the posterior covariance of that reconstruction is not able to capture the vanishing error inside the enclosed domain due to given boundary data. More severely, in contrast to the specialized GP, the posterior mean $\bar{u}$ does not satisfy Laplace’s equation $\Delta \bar{u}=0$ exactly. This leads to a violation of the classical result that (differences of) solutions of Laplace’s equation may not have extrema inside Ω, showing up in the difference to the reconstruction in Figure 3 and Figure 4. This kind of error is quantified by computation of the reconstructed charge density $\bar{q}=\Delta \bar{u}$. This is fine if data from Poisson’s equation Δu=q with distributed charges should be fitted instead. However, to keep Δu=0 exact in Ω, one requires more specialized kernels such as (27).

### 3.2. Heat Equation: Physical Parameter Estimation

Let us now consider the 1D homogeneous heat/diffusion equation over position *x* and time *t*,

(34) $$\frac{\partial u(x,t)}{\partial t}-D\Delta u\left(x,t\right)=0$$

for $\left(x,t\right)\in R\times R^{+}$. Here, the diffusivity *D* is a physical parameter determining how fast solutions spread in space. Integrating the fundamental solution

(35) $$G\left(x,t,\xi ,\tau \right)=\frac{1}{\sqrt{4\pi D(t-\tau )}}\exp\left(-\frac{{\left(x-\xi \right)}^{2}}{4D(t-\tau )}\right)$$

from ξ=−∞ to *∞* at τ=0, i.e., placing sources everywhere in space at a single initial time, and adding a scale hyperparameter $\sigma_{u}$ leads to the convolution kernel

(36) $$k_{n}\left(x,t,x^{\prime },t^{\prime };D,\sigma_{u}\right)=\frac{\sigma_{u}^{\;2}}{\sqrt{4\pi D(t+t^{\prime })}}\exp\left(-\frac{{\left(x-x^{\prime }\right)}^{2}}{4D(t+t^{\prime })}\right).$$

In terms of *x*, this is a stationary squared exponential kernel and the natural kernel over the domain x∈R. The kernel broadens with increasing *t* and $t^{\prime }$. Nonstationarity in time can also be considered natural to the heat equation, as its solutions show a preferred time direction on each side of the singularity t=0. The only difference of (36) to the fundamental solution (35) is the positive sign between *t* and $t^{\prime }$. As both *t* and $t^{\prime }$ are positive, *k* is guaranteed to take finite values and, in contrast to (35), does not become singular at $\left(x,t\right)=\left(x^{\prime },t^{\prime }\right)$.

As for the Laplace equation it is also convenient to define a non-stationary kernel by cutting out a domain that is known to be free of sources. In case heat sources are known to exist only left of the origin we evaluate the integral over the fundamental solution over (−∞,0) to

(37) $$k\left(x,t,x^{\prime },t^{\prime };D,\sigma_{u}\right)=k_{n}\left(x,t,x^{\prime },t^{\prime };D,\sigma_{u}\right)\left[1+\frac{g(x,t,x^{\prime },t^{\prime };D)}{2}\right],$$

where

(38) $$g\left(x,t,x^{\prime },t^{\prime };D\right)\equiv \mathrm{erf}\left(-\frac{x/t+x^{\prime }/t^{\prime }}{2\sqrt{D}\sqrt{1/t+1/t^{\prime }}}\right)$$

is defined via the error function erf. Choosing instead a source-free region domain interval (a,b) we integrate over R\(a,b) and obtain

(39) $$k\left(x,t,x^{\prime },t^{\prime };D,\sigma_{u}\right)=k_{n}\left(x,t,x^{\prime },t^{\prime };D,\sigma_{u}\right)\left[1-\frac{g\left(x-b,t,x^{\prime }-b,t^{\prime };D\right)-g\left(x-a,t,x^{\prime }-a,t^{\prime };D\right)}{2}\right].$$

Incorporating the prior knowledge that there are no domain sources is expected to improve the reconstruction.

**Figure 6:**
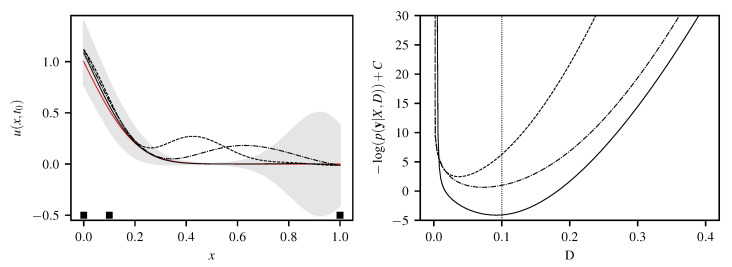
(**Left**) GP reconstruction of u(x,t=0.125) for 1D heat equation Dirichlet problem based on measurement points (▪) at x=0,0.1,1, reference in red. Kernels (36), (37) and (39) marked by dashed, dash-dotted and solid lines, respectively. 95% confidence interval bands shown only for (39), producing the best fit. (**Right**) negative log likelihood over diffusivity *D*.

As a physical example, we consider a rod with temperatures held fixed at two ends and a given initial temperature distribution. We model this as an initial-boundary value problem for (34) on the interval x∈(0,1) with Dirichlet boundary data u(0)=1 and u(1)=0. As initial conditions, we set u(x,0)=0 everywhere except at the left end where u(0,0)=1. The actual diffusivity is chosen as D=0.1, and we let u(x,t) evolve from $t_{0}=0$ until $t_{1}=1$. With increasing *t* the initial conditions are smoothed out as *u* approaches the stationary solution u(x,t→∞)=1−x. Measurements of *u* are performed at three positions x=0,0.1,1 at four times $t=10^{-5},0.25,0.5,0.75$, yielding 12 training points in total. In Figure 6 the resulting reconstruction of u(x,t=0.125) is plotted for each of the three kernels defined above. Kernel (39) allowing initial sources on both sides of the interval yields the best reconstruction. Furthermore, it is the only one that reproduces meaningful uncertainty bands based on the 95% confidence interval $\bar{u}\pm 1.96\sigma$, whereas the ones for (36) and (36) span the whole plot domain. Estimation of diffusivity *D* is also most reliable with kernel (39). The according negative log likelihood can be seen on the right plot in Figure 6. Although all three kernels produce well posed optimization problems, only (39) has the minimum at the correct position D=0.1.

The reason for the requirement of kernel (39) is clear from the statement of the problem: keeping *u* fixed on both sides of the interval can only be achieved by restricting the heat flux in a predefined way that requires sources on both sides at t=0. However, the domain itself should not contain any heat sources at any time. If we had placed an open boundary condition on the right side, kernel (37) would have been the more natural choice instead.

### 3.3. Helmholtz Equation: Source and Wavenumber Reconstruction

Finally, to demonstrate the full method, we consider the Helmholtz equation with sources:

(40) $$\Delta u\left(x\right)+k_{0}^{\;2}u\left(x\right)=q\left(x\right).$$

In 1D, solutions for the homogeneous equation with x=x are given by linear combinations of $\cos\left(k_{0}x\right),\sin\left(k_{0}x\right)$. Choosing a diagonal prior in (18) leads to a stationary kernel

(41) $$k\left(x,x^{\prime };k_{0},\sigma_{u}\right)=\cos\left(k_{0}x\right)\sigma_{u}\cos\left(k_{0}x^{\prime }\right)+\sin\left(k_{0}x\right)\sigma_{u}\sin\left(k_{0}x^{\prime }\right)=\sigma_{u}\cos\left(k_{0}\left(x-x^{\prime }\right)\right),$$

as presented in [11]. For the two-dimensional case in polar coordinates, a family of solutions based on the separation of variables is

(42) $$\cos\left(m\theta \right),\sin\left(m\theta \right),J_{m}\left(k_{0}r\right),Y_{m}\left(k_{0}r\right),$$

where $J_{m}$ and $Y_{m}$ are Bessel functions of first and second kind, respectively. Similar to the simpler 1D case, by applying Neumann’s addition theorem, we obtain a specialized kernel

(43) $$k\left(x,x^{\prime };k_{0},\sigma_{u}\right)=\sigma_{u}^{2}J_{0}(k_{0}|x-x^{\prime }\left|\right).$$

In the 3D case, one would proceed in a similar fashion with spherical Bessel functions, which yields the kernel that was already postulated in [11]. In contrast to the case of Laplace’s equation in the previous section, these source-free Helmholtz kernels do not possess singularities at any finite distance from the origin, i.e., no virtual exterior sources in the Mercer kernel (20). As a consequence they provide smoothing regularization on the order of the wavelength $\lambda_{0}=2\pi /k_{0}$ to reconstructed fields and boundary conditions that may or may not be desired. Internal sources at positions $x_{\;k}^{q}$ are linearly modeled according to (10) with basis

(44) $$h_{i}\left(x\right)=G\left(x,x_{i}^{q}\right)=H_{0}^{\left(2\right)}(k_{0}|x-x_{i}^{q}\left|\right),$$

where $H_{0}^{\left(2\right)}$ is the Hankel function of the second kind. The method of source strength reconstruction is improved compared to [11], as it constitutes a linear problem according to (15). Nonlinear optimization is instead applied to $\sigma_{u}$ and wavenumber $k_{0}$ as free hyperparameters to be estimated during the GP regression. The set-up is the same as in [11]: a 2D cavity with various boundary conditions and two sound sources of strengths 0.5 and 1.0, respectively. Results for sound pressure fulfilling (40) are normalized to have a maximum $p/p_{0}=1$. We compare three variants of GP regression for these data:

(1) Superposition of specialized kernel GP for homogeneous part $u_{h}$ and linear source model for $u_{p}$.
(2) Superposition of generic squared-exponential kernel GP for $u_{h}$ and linear source model for $u_{p}$.
(3) Generic squared-exponential kernel GP model for the full field *u*.

Naturally, after the presented analysis, only (1) can be the “correct” way of regression for this kind of data from a PDE with point sources. Variant (2) is a “hybrid” that should be able to identify point sources, while polluting the source-free part with volumetric contributions. Considering that (2) helps to separate the effect from this pollution from the effect of adding a linear source model. Variant (3) is expected to show worse performance compared to (1) and (2), as neither source-free part nor singularities of *u* at point source positions can be modeled correctly.

**Figure 7:**
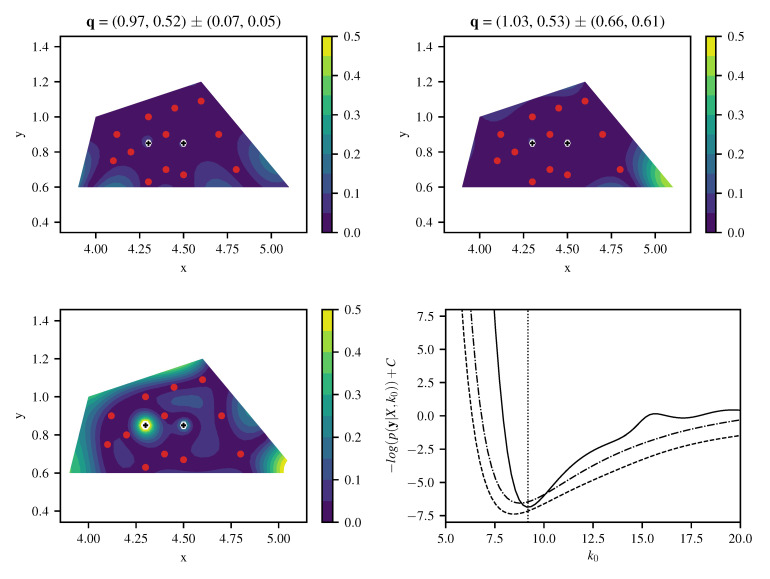
Reconstruction error for the Helmholtz equation from 12 training points for specialized kernel (**top left**), squared exponential kernel with linear source model (**top right**) and squared exponential kernel (**bottom right**); reconstructed source strengths q with 95% confidence interval via posterior (15) and (16). Negative log likelihood (**bottom right**) with optimum $k_{0}^{\mathrm{ML}}=9.19$ for specialized kernel (solid line), sq.exp. kernel with linear source model (dashed), and sq.exp. kernel alone (dash-dotted).

**Figure 8:**
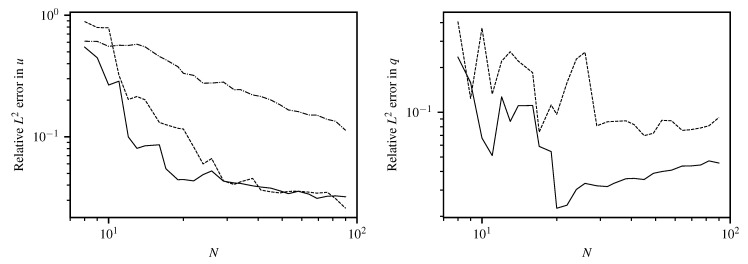
Comparison of relative $L^{2}$ error in *u* (**left**) and *q* (**right**) for specialized kernel (solid line), squared exponential kernel (dash-dotted) and squared exponential kernel with linear source model (dashed) for Helmholtz equation with *N* quasi-random training points. As the squared exponential kernel alone (without linear source model) cannot reproduce point sources, no result is shown for the point source strength estimation in the right plot for this case.

Figure 7 shows the local absolute field reconstruction error based on 12 training data points with artificial noise of $\sigma_{n}=0.01$. Hyperparameters $k_{0}$ and $\sigma_{u}$ are set to optimized ML values, and $\sigma_{n}$ is fixed to its actual value. The upper left plot shows results for variant (1) with the specialized kernel (43). Variant (3) with a generic squared exponential kernel (33) of length scale $\ell =\pi /(\sqrt{2}k_{0})$ to model *u* yields a much higher field reconstruction error as depicted in the lower left of Figure 7. The field reconstruction using the generic kernel is improved when a linear model for the inhomogeneous term is included (variant (2)), as shown in the upper right of Figure 7. However, the original differential Equation (40) is only fulfilled exactly when using a specialized kernel with $\hat{L}k\left(x,x^{\prime }\right){\hat{L}}^{\prime }=0$. As expected, variant (1) produces the best reconstruction at a given number of training points (Figure 8). There the first 12 points are chosen as marked in Figure 7, and more points are generated from a quasi-random Halton sequence. The obtained negative log-likelihood (Figure 7, lower right) depending on $k_{0}$ and $\sigma_{u}$ at its ML value demonstrates the well-posedness of estimating $k_{0}$ having the physical meaning of a wavenumber. Variants (2) and (3) lead to a slightly less peaked estimate for a spatial length scale hyperparameter without a direct physical interpretation.

**Figure 9:**
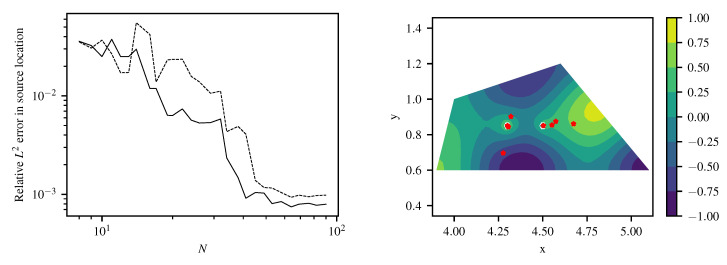
(**Left**) Comparison of relative $L^{2}$ error in source position for specialized kernel (solid line) and squared exponential kernel with linear source model (dashed) for Helmholtz equation with *N* quasi-random training points. (**Right**) reconstructed field using specialized kernel (43) and showing convergence of estimated source location for N=(12,15,20,30) quasi-random training points.

For estimation of source positions, nonlinear optimization is applied to source positions as free hyperparameters within the given boundaries, employing an evolutionary algorithm CMA-ES [17]. The results of source strength and position estimation using (15) and (16) in the configuration with 12 training points is given in Table 1. Both estimates match the exact values reasonably well. At an increasing number of training data the reconstruction becomes more accurate, stagnating at an error between 0.1% and 1% and showing the advantage of the specialized kernel more clearly (Figure 8 and Figure 9). The relative $L^{2}$ error in source positions for specialized and generic squared exponential kernel with linear source model is depicted in the left plot of Figure 9. Again, results from the specialized kernel are usually more accurate and stable compared to using a squared-exponential kernel for the source-free part of the field at a given number of training points.

## 4. Summary and Outlook

A framework for application of Gaussian process regression to data from underlying linear partial differential equations with localized sources has been presented. The method is based on superposition of a Gaussian process that generates exact solutions of the homogeneous equation, complemented by a linear model for sources. For the homogeneous part, specialized kernels are constructed from fundamental solutions via Mercer’s theorem. For source contributions, fundamental solutions are used as basis functions in the linear model. Examples for suitable kernels have been given for Laplace’s equation, heat equation and Helmholtz equation. Regression has been shown to yield better results compared to using a squared exponential kernel at the same number of training points in the considered application cases. Advantages of the specialized kernel approach are the possibility to represent exact absence of sources as well as physical interpretability of hyperparameters. This comes at the cost of requiring non-standard, possibly nonstationary kernels. The presented method has been demonstrated to be able to accurately estimate system parameters such as diffusivity and wavenumber, as well as position and strength of point sources using only around 10 training data points in two-dimensional domains.

In a next step, reconstruction of vector fields via GPs could be formulated, taking laws such as Maxwell’s equations or Hamilton’s equations of motion into account. A starting point could be squared exponential kernels for divergence- and curl-free vector fields [18]. Such kernels have been used in [19] to perform statistical reconstruction, and [20] apply them to GPs for source identification in the Laplace/Poisson equation. To model Hamiltonian dynamics in phase-space, vector-valued GPs could possibly be extended to represent not only volume-preserving (divergence-free) maps but retain full symplectic properties, conserving all integrals of motion such as energy or momentum.

## Figures and Tables

**Table 1:** Comparison and results for estimation of source strength q and source position $x_{i}^{q}$ for 12 training data points for specialized and squared exponential kernel with linear source model.

Exact Values	Specialized Kernel	sq. exp. Kernel
q=(1.0,0.5)	q=(0.97,0.52)	q=(1.03,0.53)
$x_{1}^{q}=\left(4.3,0.85\right)$	$x_{1}^{q}=\left(4.31,0.85\right)$	$x_{1}^{q}=\left(4.30,0.82\right)$
$x_{2}^{q}=\left(4.5,0.85\right)$	$x_{2}^{q}=\left(4.65,0.90\right)$	$x_{2}^{q}=\left(4.61,0.84\right)$
